# Motor Competence and Physical Activity in Early Childhood: Stability and Relationship

**DOI:** 10.3389/fpubh.2020.00039

**Published:** 2020-02-21

**Authors:** Einat A. Schmutz, Claudia S. Leeger-Aschmann, Tanja H. Kakebeeke, Annina E. Zysset, Nadine Messerli-Bürgy, Kerstin Stülb, Amar Arhab, Andrea H. Meyer, Simone Munsch, Jardena J. Puder, Oskar G. Jenni, Susi Kriemler

**Affiliations:** ^1^Epidemiology, Biostatistics and Prevention Institute, University of Zurich, Zurich, Switzerland; ^2^Child Development Center, University Children's Hospital Zurich, Zurich, Switzerland; ^3^Children's Research Center, University Children's Hospital Zurich, Zurich, Switzerland; ^4^Department of Clinical Psychology and Psychotherapy, University of Fribourg, Clinical Psychology and Psychotherapy, Fribourg, Switzerland; ^5^Endocrinology, Diabetes & Metabolism Service, Centre Hospitalier Universitaire Vaudois (CHUV), Lausanne, Switzerland; ^6^Department of Psychology, University of Basel, Basel, Switzerland; ^7^Division of Pediatric Endocrinology, Diabetology and Obesity, Centre Hospitalier Universitaire Vaudois (CHUV), Lausanne, Switzerland

**Keywords:** motor competence, fundamental movement skills, physical activity, children, preschool, longitudinal, splashy

## Abstract

**Background:** Normal motor development and adequate levels of physical activity engagement during the early years of life form the foundation of long-term psychological and physiological health. This is one of the very few studies that investigate the stability and relationships of motor competence and physical activity in preschool children.

**Methods:** Baseline and 12 month follow-up data of physical activity and motor competence of 550 preschool children aged 2–6 years from the Swiss Preschoolers' Health Study were used for this work. Physical activity data, expressed in counts per minute for total physical activity and minutes per day for time spent moderately-to-vigorously physically active, were collected over 1 week using accelerometers. Motor competence was assessed with the Zurich Neuromotor Assessment. Both motor competence and physical activity were age- and sex-adjusted. To examine the individual stability of physical activity and motor competence and reciprocal cross-sectional and longitudinal effects between these two domains, a latent variable cross-lagged panel model where motor competence was represented through a latent construct was examined using structural equation modeling.

**Results:** A weak cross-sectional correlation of motor competence with total physical activity (*r* = 0.24) and moderate-to-vigorous physical activity (*r* = 0.23) was found. Motor competence exhibited high stability (β = 0.82) in the preschool years and physical activity was moderately stable with estimates ranging from β = 0.37 for total physical activity to β = 0.48 for moderate-to-vigorous physical activity. In contrast to the autoregressive coefficients denoting individual stability, both cross-lagged effects were negligible indicating that physical activity was not a determinant of motor competence or vice versa.

**Conclusions:** Motor competence and physical activity developed independently of each other in early childhood. Although measures of quantity and intensity of physical activity were not related to motor development, specific movement experiences and practice—which are not reflected by accelerometry—may be needed for skill development. Future research should focus on examining what type of physical activity is important for motor development and how to assess it, and also whether the relationship between physical activity and motor competence evolves over time.

**Clinical Trial Registration:** Current Controlled Trials ISRCTN41045021 (date of registration: 21.03.14)

## Introduction

Both physical activity (PA) and motor competence (MC) have been linked to improved health indicators including increased cardiorespiratory fitness and decreased adiposity (1–3). MC is a global term used to describe goal-directed gross movements that involve large muscle groups or the whole body (e.g., running, jumping, balancing) (3). In early childhood, children begin to learn how to move their body through space by developing so called fundamental movement skills, which form the foundation for future more complex movement skills. Motor development is an iterative learning process driven by changes in the structure or function of the body as well as the environment (4, 5). There is increasing agreement about the existence of a continuous interplay of nature and nurture in defining motor development. Theories have moved away from neuro-maturational approaches claiming a predetermined sequence of motor skill acquisition (6) to a more holistic view involving contextual and biological factors (5, 7). From the dynamic systems perspective motor skills develop in a perpetual interplay between the organism, environment and task constraints, which may vary across stages of development. PA, an important element of this complex system, is a behavior that is needed to attain or improve MC. At the same time it can be seen as a product of motor development. This chicken-and-egg dilemma has been feeding the debate about how MC and PA are related over decades (8, 9).

Since evidence suggests that behavioral capabilities and lifestyle habits establish in childhood and track over time (10, 11), appropriate development of MC and levels of PA are important not only for child health, but also to sustain health throughout life. Numerous researchers have investigated underlying pathways including inter- and intra-individual variation and relationships. While cross-sectional evidence indicates a positive association between MC and PA in children and youth (12–14), only a few longitudinal studies investigating the suspected causal pathways between MC and PA have been published (15–19). Studies in older children found MC and self-reported PA to be unrelated (20), MC to be predictive of subsequent self-reported PA (16, 17), objectively measured PA to be predictive of subsequent MC (21) or a reciprocal longitudinal relationship between MC and self-reported (18) or objectively measured (19) PA. The only study focusing specifically on preschool children showed that objectively measured moderate-to-vigorous PA at 3.5 years, but not at 19 months, was predictive of locomotor skills at age 5 (15).

Thus, current evidence relies on predominantly cross-sectional studies that show overall small effects. Whether a real causal pathway exists, and if so, whether it is unidirectional or reciprocal is unclear. To better understand how PA and MC are related in early childhood, more longitudinal studies that allow for cause-effect pathways in both directions are needed. The concept of reciprocal influence was first described in 2008 by Stodden et al. (8). The authors developed a theoretical framework where the direction of causation was hypothesized to change from early to middle childhood. In young children, PA was suggested to drive the development of MC through a variety of exploratory and structured movement experiences that promote neuromotor development. As children transition to middle and late childhood, the relationship was hypothesized to become stronger and more reciprocal, driven by the child's ability to perceive its competence in various movement contexts.

To the best of our knowledge, no longitudinal study has investigated the stability and reciprocal relationship of objectively measured PA and MC focusing specifically on preschool children. Such studies are important not only from a public health perspective, enabling the design of more effective and properly timed preventive measures or interventions that ultimately inform guidelines and recommendations. Also on the individual level, a thorough understanding of the stability and interplay can help predict performance and identify children with mild to severe delay or impairment of motor development requiring specialized clinical intervention during this crucial window of early childhood. Thus, to move research forward we investigated (a) the cross-sectional association between MC and PA, (b) the individual stability over time and (c) the longitudinal reciprocal relationship of MC and PA in preschool children. Because some evidence suggests that besides quantity of PA the intensity may affect the relationship between PA and MC, we used two different constructs of PA [total PA (TPA) and moderate-to-vigorous PA (MVPA)] (2). We also examined a potential moderating effect of sex and age group (younger vs. older) on the aforementioned research questions.

## Materials and Methods

### Study Population

Data presented in this work are drawn from the Swiss Preschoolers' Health Study (SPLASHY; ISRCTN41045021), a multi-site prospective cohort study including 555 2- to 6 year-old preschool-aged children from 84 childcare centers located in five cantons of Switzerland (covering 50% of the Swiss population in 2013). Sampling of childcare centers was stratified according to one stratum with four levels: urban community and rural community with high socio-economic status (SES; above-average) and low SES (below-average), each based on the prevalence of child care centers in the respective communities. In total, 639 child care centers were contacted between January 2013 and October 2014, of which 126 child care centers agreed to participate and to inform the parents. Forty-two centers were excluded after the preparation of testing dates due to too few (less than two) participating children (78%) or for other reasons (12%). Data collection in childcare centers was conducted in 2014 and 1 year later by the same study team in parallel at all study sites. Children recruited in 2014 (*n* = 476) had a follow-up assessment 1 year later. Those recruited in 2015 (*n* = 79) had a baseline assessment only. Both baseline (T0) and follow-up (T1) data are used in the current study. Ethical approval is in accordance with the Declaration of Helsinki and has been obtained from all local ethical committees (No 338/13 for the Ethical Committee of the Canton of Vaud as the main approving authority). Children and parents provided oral and written informed consent. A detailed description of the study design has been published elsewhere (22).

### Measures

#### Physical Activity

PA was objectively monitored on seven consecutive days using a hip-worn accelerometer (wGT3X-BT, ActiGraph, Pensacola, FL, USA). Participants were instructed to wear the monitor 24 h/day except during water-based activities. A sampling frequency of 30 Hz was used. Accelerometer data were downloaded in 3-s epochs and aggregated to 15 s epochs. Nighttime hours (9 pm to 7 am) and non-wear periods, defined as ≥20 min of consecutive zero counts on all axes (23), were excluded. A monitoring day was considered valid if at least 10 h of activity were recorded. PA outcome data were expressed as counts per minute [cpm] for TPA and min/day for MVPA (defined as ≥420 counts per 15 s) (24). Since at both time points TPA and MVPA did not differ between participants who provided at least 3 valid days including 1 weekend day (baseline: 91 %, follow-up: 89 %), and those with less days of recording, all participants were included in analysis.

#### Motor Competence

After measuring height and body weight by standard procedures, MC was assessed using the Zurich Neuromotor Assessment 3–5 (ZNA 3-5) (25, 26), which is based on the original ZNA for children older than 5 years (ZNA 5–18) (27, 28). The ZNA 3-5 is a well-standardized motor test instrument with good intra-observer (0.56–1.00) and inter-observer (0.42–0.99) reliability, while test-retest reliability is lower in some tasks (0.35–0.84) (25). Five components were used to capture gross motor proficiency: static balance, walking on a straight line, sideward jumping, hopping on one leg and running. All tasks were videotaped, which allowed offline rating. The examiner explained and demonstrated each task. If children did not understand the task or did something different, the demonstration was repeated. In case of a second failure, the examiner scored the task as “failed” and continued the assessment. Instructions for the tasks were as follows: (1) Static balance: Children were asked to do a one-leg stand for as long as possible. Time counting started as soon as the child lifted one foot off the floor and stopped when the child touched the floor with the lifted foot or shifted the foot of the standing leg more than 2 cm. The same procedure was repeated for the other leg. A qualitative score from 0 to 4 was given: 0 = one-leg stand more than 5 s on both legs; 1 = one-leg stand more than 5 s on only one leg; 2 = one-leg stand between 2 and 5 s on both legs; 3 = one-leg stand between 2 and 5 s on only one leg; 4 = not able to stand on either leg for more than 2 s. (2) Walking on a straight line: Children were asked to walk on a straight line consisting of an elastic band placed on the floor putting one foot in front of the other such that the heel of the front foot touched the toes of the back one. Rating included a qualitative score from 0 to 4: 0 = perfect performance, heel touches toes; 1 = feet straight on the line but gap between the feet; 2 = feet not straight and/or off the line up to 3 times; 3 = feet perpendicular and/or feet off the line more than 3 times; 4 = not able to walk on the line. (3) Jumping sideways: Children were asked to jump sideways over the elastic band back and forth keeping the feet together. Rating included a qualitative score from 0 to 4: 0 = perfect performance, very smooth jumping; 1 = jumping performed correctly but not very smoothly; 2 = touchdown with both feet at the same time, stiff movements; 3 = total body involvement, poor coordination in relation to the band direction; 4 = Jumping over the elastic band but not in relation to the band direction. (4) Hopping on one leg: Children were asked to hop as many times as possible on one leg. Two trials per leg were given. Rating included a qualitative score from 0 to 4: 0 = hopping on both legs more than 7 times; 1 = hopping on only one leg more than 3 times; 2 = hopping on both legs up to 3 times; 3 = hopping on only one leg 1–3 times; 4 = cannot hop on either leg. (5) Running: Children were asked to run around the cord (at least 20 meters). Rating included a qualitative score from 0 to 4: 0 = rolling motion of feet with adjustment of upper body; 1 = rolling motion of feet, stiff upper body; 2 = running with partial rolling motion of feet; 3 = running without rolling motion of feet; 4 = cannot run (no flight phase).

### Statistical Analyses

Statistical analyses were performed using R version 3.4.4 (R Foundation for Statistical Computing, Vienna, Austria). Descriptive statistics are presented as mean [standard deviation [SD]] and ranges for continuous variables and percentages for categorical variables, unless stated otherwise. Participants without PA and MC data were excluded from the analysis (n = 5), resulting in a sample of 550 individuals. Q-Q plots and frequency distributions were used to check for normal distribution and potential outliers. MC measures were standardized and expressed as standard deviation scores calculated from age- and sex-adjusted normative values to receive identical metrics across tasks. PA was also age- and sex-standardized to account for known age and sex effects. Thus, positive values correspond to above average performance or PA, respectively, and negative values indicate below average measurements within the same sex and age group. To investigate the hypothesized reciprocal longitudinal relationship between MC and PA, a latent variable cross-lagged panel model using structural equation modeling was created, where MC was represented through a latent construct (29). The latent constructs were first verified as separate measurement models with confirmatory factor analysis (CFA). The CFA and latent variable cross-lagged panel model were performed using the package lavaan (30). Full information maximum likelihood (FIML) was used to handle missing data and results were compared to a complete case analysis (*n* = 218). FIML is known to lead to unbiased estimates if the data are either missing completely at random (MCAR) or missing at random (MAR) whereas complete case analysis requires MCAR for unbiased estimates and suffers from reduced power due to reduction in sample size. In line with previous recommendations, good model-data fit was characterized by a non-significant χ2-test statistic, a standardized root mean square residual (SRMR) <0.08, a root mean square error of approximation (RMSEA) <0.06, and a comparative fit index (CFI) >0.90 (31). Bootstrapping was used to ensure robustness of model fit indices (500 bootstrap replications) (32, 33). Estimated paths were adjusted for accelerometer wear time. Additional analyses were conducted to further evaluate the robustness of our findings: (1) Multigroup structural equation modeling was applied to investigate whether sex or age group (younger vs. older children by using the sample median) moderated the relationship between PA and MC, (2) Estimated paths were additionally controlled for SES and excess body weight to exclude potential confounding. SES was assessed using the International Socio-Economic Index of occupational status (ISEI) (34, 35), which assigns values between 16 (manual labor in agricultural sector) and 90 (judge) to job titles with respect to education and income. BMI z-scores, calculated based on the World Health Organization growth charts were used as an indicator for body fatness. The statistical significance level alpha was set at 0.05.

## Results

Descriptive statistics are shown in Table 1. The sample comprised 550 preschool children (47% boys) aged 3.9 (0.7) years at baseline and 4.9 (0.7) at follow-up. Comparisons between baseline and follow-up measurements demonstrated that on average children performed better in MC tests and were more physically active at follow-up (all *p* < 0.05, paired *t*-tests). All children met the guidelines of at least 180 min of any PA per day at both time points (37–39). There were no differences in baseline demographic characteristics (age, SES, BMI) between children with complete and incomplete data (data not shown). Both MC measurement models (baseline and follow-up) showed a good fit; χ2(5) = 0.59, *p* = 0.99; SRMR = 0.01; RMSEA = 0.00; CFI = 1.00 and χ2(5) = 1.16, *p* = 0.76; SRMR = 0.01; RMSEA = 0.00; CFI = 1.00. Similarly, the latent variable cross-lagged panel models with TPA [χ2(45) = 41.05, *p* = 0.47; SRMR = 0.02; RMSEA = 0.00; CFI = 0.99] and MVPA [χ2(45) = 39.11, *p* = 0.46; SRMR = 0.03; RMSEA = 0.00; CFI = 0.99] demonstrated good overall fit. No *post-hoc* modifications were conducted.

**Table 1:** Characteristics of participants at baseline and follow-up (*n* = 550).

	**Baseline**		**Follow-up**	
	**Mean (SD)**	**Range**	**Mean (SD)**	**Range**
Age (years)	3.9 (0.7)	2.2–6.6	4.9 (0.7)	3.2–7.6
BMI z-score^a^	0.4 (1.0)	−4.0–4.7	0.3 (0.9)	−3.3–5.4
SES	62.9 (15.5)	17.0–89.0	62.5 (15.5)	17.0–89.0
Monitor wear time [h/day]	12.8 (0.7)	10.2–14.0	13.0 (0.9)	10.2–14.0
TPA [cpm]	623 (153)	243–1,331	643 (154)	260–1,797
MVPA [min/day]	92 (29)	26–206	97 (28)	19–201
Walking^b^	2.2 (0.8)	0–4	1.8 (0.8)	0–4
Jumping^b^	2.6 (1.0)	0–4	2.0 (1.2)	0–4
Hopping^b^	2.4 (1.4)	0–4	1.2 (1.3)	0–4
Running^b^	2.4 (0.8)	0–4	1.9 (0.6)	0–4
Static balance^c^ [sec]	8.0 (8.0)	2–75	12.4 (10.4)	2–88

a *Based on WHO growth standards (36)*.

b *Measured on an ordinal scale, lower scores indicate better performance*;

c *based on dominant leg, longer duration indicates better performance; BMI, Body mass index; SES, Socioeconomic status; TPA, Total physical activity; cpm, counts per minute; MVPA, moderate-to-vigorous physical activity*.

**Figure 1:**
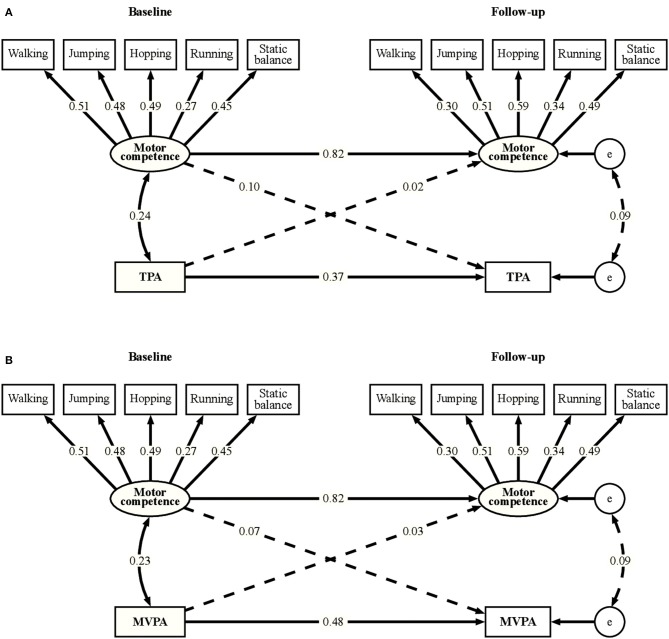
Latent variable cross-lagged panel models for total physical activity **(A)** and moderate-to-vigorous physical activity **(B)**. TPA, Total physical activity; MVPA, moderate-to-vigorous physical activity; e, error term. Solid lines indicate paths significant at *p* < 0.05, non-significant paths are presented as dashed lines. Numbers are standardized coefficients. Correlations between residuals of indicator variables are not shown for simplicity.

Figure 1 depicts the latent variable cross-lagged panel models for TPA (A) and MVPA (B), respectively, for the whole group. MC was highly stable over time with an autoregressive coefficient of β = 0.82 (*p* < 0.001). Both TPA and MVPA showed moderate stability, autoregressive coefficients were β = 0.37 (*p* < 0.001) and β = 0.48 (*p* < 0.001), respectively. Cross-lagged coefficients between PA and MC were very small and not significant [standardized coefficients for the effect of TPA at baseline on MC at follow-up: β = 0.02 (*p* = 0.77), for the effect of MC at baseline on TPA at follow-up β = 0.10 (*p* = 0.25)]. A significant but weak cross-sectional association of MC with TPA and MVPA was found at baseline [*r* = 0.24 (*p* < 0.001) and *r* = 0.23 (*p* = 0.001), respectively]. Multigroup analyses revealed that neither age group nor sex had a moderating effect on the relationships between MC and PA in the model. Furthermore, adjusting for SES and BMI z-score did not change the observed effects.

## Discussion

To the best of our knowledge, this is the first study to examine both the stability and reciprocal relationship of MC and PA within a relatively large cohort of preschoolers using objective measures. We found that at this young age, children demonstrated diverse levels of MC and PA that were unrelated. Individual stability, i.e., the tendency to maintain the same relative position within a cohort, was relatively high for MC and moderate for PA over a 1 year period. Our findings suggest that MC and PA develop independently of each other and track over time during the period of early childhood.

Although we found a significant but weak cross-sectional association between PA and MC, analysis of cross-lagged effects indicated that PA was not predictive of MC in early childhood or vice versa. Previous cross-sectional studies in preschoolers found similar results (21, 40, 41). A study of 394 3–5 year-old children (40) reported slightly lower but significant positive correlations of TPA (*r* = 0.10) and percent time in MVPA (*r* = 0.18) with total movement skills. Others found similar correlations with total movement skills (MVPA, *r* = 0.20; VPA, *r* = 0.26) (41) and dynamic balance skills (TPA, *r* = 0.20; MVPA, *r* = 0.22) (21). The only study examining the longitudinal relationship in young children (*n* = 185) found that MVPA at 3.5 years predicted locomotor skills, but not object control skills or total skill competence, at age 5 (15). Investigations in older children reported mixed results (16–20), which further illustrates that the nature and strength of the relationship may not be straightforward. A possible explanation for our null result is the way through which PA was operationalized. Although habitual PA *per se* may be important for some health-related outcomes, it may not promote MC. Specific types or quality of movement experiences may be required rather than overall movement quantity or intensity. High participation in balancing activities for example would not be represented by high levels of total or moderate-to-vigorous PA, yet would likely be associated with better balancing competence. This idea has previously been confirmed by a study investigating participation in different physical education activities in preschool aged children (42). Similarly, the implementation of planned movement programs was found to be effective at improving MC as compared to “free play” (43, 44). Along the same lines, it is important to consider how MC has been operationalized when analyzing the relationship with PA and comparing results. A systematic review of motor skill correlates found that only some aspects of MC were related to PA (14). While skill composite and motor coordination had a positive association with PA, evidence was indeterminate for object control and locomotor skills. Lastly, the use of different PA assessment tools (self-report, direct observation, or accelerometry) and MC instruments (quantitative vs. qualitative assessment batteries) as well as analysis approaches of PA [choice of epoch length and intensity cut-points (45)] can greatly impact the results.

We further hypothesized that the relationship between MC and PA strengthens as children age. As children transition to middle childhood, the sum of all influences is thought to lead to a positive or negative spiral of engagement that compounds over time (8) and affects health-related risk factors. Individuals with low actual and perceived MC for example will be drawn into a negative spiral of disengagement resulting in reduced sport involvement, low levels of PA or even obesity (8). While 70% of the children in our sample were below the age of 4 at baseline and potentially primarily constrained by endogenous developmental steps of maturation (14), we hypothesized that additional physiological or psychological factors, such as children's perceptions of their own MC, may develop at 5–6 years and influence the relationship. Yet, we did not find the relationship to strengthen as children aged. We assume that a longer time span beyond the fundamental movement skills development period would be required to capture the expected increase in strength of association (8).

Our findings indicated that MC was stable over time during the preschool period whereas PA exhibited moderate stability. Somewhat lower stability coefficients were found for MC in a longitudinal study from 4 to 5 and 6 years (boys: 0.58–0.69, girls: 0.31–0.47; *N* = 205) (11) and similar coefficients were found for PA (10, 46). It is plausible that the lower stability coefficients of PA compared to MC in early childhood are due to different etiological pathways and adaptability to internal and external factors. Habitual PA as a behavior may entail more flexibility and thus greater variation than a construct that at this stage is, at least in part, influenced by biological maturation (7).

Previous literature has not offered a clear answer as to whether the stability and relationship of PA and MC differed by sex (13). Some authors found different developmental trajectories between the two sexes, particularly in later childhood and adolescence, such that girls were more proficient in locomotion components whereas boys scored better in object control tasks (47–50). The MC assessment battery we used did not assess object control/manipulation skills, which could be a possible explanation of why we did not find a moderating effect of sex. However, this difference has often been found in middle childhood and adolescence and basic patterns were shown to be similar in boys and girls during the preschool period (11, 14). This suggests that various opportunities for practice may impact the development of specific skills (14, 43) and that opportunities and motivation for engagement in different context- or skill-specific activities may vary for boys and girls.

Important strengths of this study include (a) the longitudinal panel design, which allowed drawing some conclusions on change over time and direction of association, (b) the relatively large cohort of children from 2 to 6 years covering the whole preschool range, (c) the fact that PA was objectively assessed and MC was videotaped and rated offline by experts and (d) the use of state-of-the-art statistical methods to model latent constructs and test complex reciprocal relationships. Limitations that need to be addressed when interpreting our results include the short follow up time, the fact that not the entire range of motor skills was assessed (e.g., no object control) and that accelerometers likely underestimate PA in preschoolers as water-based and rolling activities are insufficiently reflected (23). Another potential weakness is that MVPA was defined using fixed cut-offs, which has a high probability of misclassification of MVPA because individual variation in such cut-offs. We are confident that taking another cutoff for MVPA would not change the result of the paper as we have also presented data with similar results on total PA that provides a safer way. Although we do not know whether children who do not attend childcare behave differently, the generalizability of our findings may be limited by the fact that our sample included only children who attended childcare at least twice a week.

This is the first longitudinal study that provides evidence on the stability and reciprocal relationship of PA and MC in young children. Based on our findings MC is a stable construct that is not influenced by the quantity or intensity of habitual PA at this early age. To inform the design of effective interventions, future longitudinal studies should examine what type of physical activity is important for motor skill development, how to assess it, and whether the (reciprocal) relationship between PA and MC evolves as children age.

## Ethics Statement

Ethical approval is in accordance with the Declaration of Helsinki and has been obtained from all local ethical committees (No 338/13 for the Ethical Committee of the Canton of Vaud as the main approving authority). Children and parents provided oral and written informed consent.

## Author Contributions

Conceived and designed the Splashy study: JP, SK, SM, and OJ. Performed data collection: ES, CL-A, AZ, TK, KS, NM-B, and AA. Designed the research, had full access to all data, and take responsibility for the integrity of data and accuracy of data analysis: ES and SK. Assisted in statistical data analysis: AM. Performed data analysis and wrote, reviewed, and edited the manuscript: ES. All authors reviewed, edited, and approved the manuscript.

### Conflict of Interest

The authors declare that the research was conducted in the absence of any commercial or financial relationships that could be construed as a potential conflict of interest.

## Abbreviations

BMI: body mass index
CFA: confirmatory factor analysis
CFI: comparative fit index
cpm: counts per minute
FIML: full information maximum likelihood
ISEI: international socio-economic index
MAR: missing at random
MC: motor competence
MCAR: missing completely at random
min: minute
MVPA: moderate-to-vigorous physical activity
PA: physical activity
RMSEA: rfoot mean square error of approximation
SD: standard deviation
SES: socio-economic status
SRMR: standardized root mean square residual
TPA: total physical activity.
